# Role of Caveolin-1 in Inflammation: Genetic Predisposition and Potential Implication for Multiple Sclerosis

**DOI:** 10.3390/genes17050593

**Published:** 2026-05-21

**Authors:** Bruk Getachew, Matthew R. Miller, Harold E. Landis, Robert E. Miller, Yousef Tizabi

**Affiliations:** 1 Department of Pharmacology, Howard University College of Medicine, Washington, DC 20059, USA; bruk.getachew@howard.edu; 2 NutriGenetic Research Institute, Ephrata, PA 17522, USA; matthewm@tolhealth.com (M.R.M.); bobm@tolhealth.com (R.E.M.); 3 Integrative Medicine Fellow, University of Arizona, Tucson, AZ 85724, USA; heldds83@gmail.com

**Keywords:** Caveolin-1, caveolae, inflammation, autophagy, oxidative stress, BBB, ICAM, MS, systems biology, *HLA-DR15*, genetic model

## Abstract

Multiple Sclerosis (MS) is a chronic, immune-mediated disorder of the central nervous system characterized by leukocyte infiltration, inflammation, demyelination, and progressive neurodegeneration. Susceptibility to MS is influenced by genetic factors, including variants within the human leukocyte antigen (*HLA*) region, (notably *HLA-DR15*), and multiple single nucleotide polymorphisms that modulate T cell function and immune regulation. Clinically, early manifestations such as visual disturbances, sensory deficits, fatigue, and impaired coordination often precede more advanced features, including cognitive decline and bladder or bowel dysfunction. Although experimental and genetic models of neuroinflammation have facilitated the development of therapies that reduce relapse rates and slow disease progression, the underlying pathological mechanisms remain incompletely understood. Emerging evidence points to the importance of cytoskeletal organization and membrane-associated signaling platforms in maintaining neuronal and immune cell function. Disruption of these systems may contribute to demyelination and neuroinflammatory cascades. Within this context, a systems biology perspective is particularly valuable, as it emphasizes the integration of multiple, interdependent pathways rather than isolated mechanisms. Caveolin-1 (Cav-1), an integral membrane protein of caveolae, has gained attention as a potential central regulator due to its role in coordinating signaling processes across diverse cellular compartments. In this review, we examine the potential genetic and functional contributions of Cav-1 to MS pathophysiology, with a focus on its involvement in oxidative stress, inflammation, blood–brain barrier integrity, and autophagy. By framing these processes as components of an interconnected network, we highlight Cav-1 as a context-dependent modulator that may influence both disease progression and severity. However, despite its mechanistic relevance, the translational potential of Cav-1 remains uncertain, and further studies are required to clarify its precise role and evaluate its suitability as a therapeutic target in MS.

## 1. Introduction

### 1.1. Systems Biology—Cav-1

Systems biology (SB) is an interdisciplinary approach to study how components of biological systems interact at DNA, RNA, and protein levels, to produce collective behavior of a system. It measures the organization and dynamics of a system to get insights into the functioning of living organisms [1]. In this regard, SB refers to an integrative, multi-level perspective that links molecular, cellular, and physiological processes, rather than implying the use of large-scale computational modeling or high-throughput data analysis. SB can help streamline efforts to understand pathway interactions between inflammation and neurodegenerative diseases by uncovering pathophysiology, early diagnostic biomarkers, and novel therapeutic targets [2]. Genome-wide association studies (GWAS) have transformed the field of multiple sclerosis (MS), leading to the identification of over a hundred risk loci. However, integrating this extensive genetic information into a unified biological framework that clarifies the underlying causes remains a significant challenge. Given the heterogeneity of MS and the inherent complexity of the human genome, several rational strategies can be proposed to better understand the biological functions associated with MS susceptibility and pathophysiology. In this context, SB can utilize computational algorithms to identify potential sub-networks. For example, SB explores the characteristics of the neuronal cytoskeleton to provide insights into how genes and their alterations can lead to MS [3]. Thus, it may prove crucial in developing novel prevention and/or treatment options. Indeed, SB can enhance personalized medicine by customizing MS interventions to individual molecular and genetic profiles [4,5].

SB, as an integrative, multi-level perspective that links molecular, cellular, and physiological processes, positions caveolin-1 (Cav-1), an integral structural protein forming caveolae, as a central regulatory hub in MS. Rather than acting in isolation, Cav-1 can be viewed as coordinating crosstalk between inflammation, blood–brain barrier (BBB) integrity, oxidative stress, and autophagy. For instance, Cav-1-mediated modulation of inflammatory signaling may influence endothelial function and BBB permeability, enabling immune cell infiltration into the central nervous system (CNS). This infiltration amplifies local inflammation and promotes oxidative stress, which in turn can disrupt cellular homeostasis and trigger or impair autophagic pathways. Dysregulated autophagy may then further enhance inflammatory responses and cellular damage, creating a self-reinforcing cycle. Positioning Cav-1 within this interconnected network helps unify these previously thought to be independent processes. Moreover, it supports Cav-1 consideration as a dynamic disease modifier operating across multiple biological systems rather than a single-pathway factor.

Herein, we employ a conceptual systems-level approach to examine the potential role of the Cav-1 in regulating autophagy, inflammation, and BBB integrity in neurodegenerative diseases, in general, and in MS, in particular. Cav-1 is an essential structural component of caveolae—small, flask-shaped invaginations of the plasma membrane—where it plays a critical role in cellular signaling. Notably, genetic alterations in Cav-1 have been implicated in increased MS susceptibility [6], highlighting its potential as a novel therapeutic target in this debilitating autoimmune disorder.

### 1.2. Multiple Sclerosis

#### 1.2.1. Etiology

MS is the most recognized autoimmune demyelinating disease of the CNS typified by chronic neuroinflammation, lymphocyte infiltration, myelin destruction, gliosis, varying degrees of axonal and oligodendrocyte pathology, and progressive neurological dysfunction [7,8,9]. In addition, the infiltration of peripheral autoreactive immune cells into the CNS is accompanied by the activation of innate immune mechanisms [10,11,12]. MS remains the leading cause of non-traumatic disability in young adults between 20 and 30 years of age [13]. Its etiology remains unclear and there is no cure [9]. Currently, the two proposed hypotheses on etiology of MS include: 1. the “outside-in” model which proposes that an aberrant autoimmune response initiated in the periphery leads to CNS damage, and 2. the “inside-out” model which posits that a primary neurodegenerative process within the CNS triggers a secondary immune response against myelin debris [14]. It is clear now that both mechanisms contribute to MS initiation and progression albeit differing primarily in their temporal sequence [15].

Once structural and functional brain alterations take place, they eventually lead to cognitive, sensory, and motor impairments [9,16,17]. The clinical presentations can vary widely among patients ranging from severe neurological defects to motor disabilities [18]. There are four types of MS, namely, clinically isolated syndrome (CIS), relapsing-remitting MS (RRMS), secondary progressive MS (SPMS), and primarily progressive MS (PPMS). CIS is a single episode of neurologic symptoms that lasts 24 h or longer. MS progression also varies among individuals transitioning from one form of RRMS to SPMS [19]. Unlike SPMS, PPMS progresses slowly yet steadily. Neurodegeneration and inflammation occur in all four types of MS, but in contrast to CIS, RRMS is characterized by the onset of symptoms that can last longer than 24 h per episode, [20] followed by a period of clinical remission [21,22]. RRMS is the most prevalent form of the disease, affecting 85% of patients [23]. Early symptoms manifest as blurred/double vision, numbness, fatigue, and balance coordination, which progress to include cognitive as well as bladder and bowel dysfunction [24].

Despite substantial advances in defining the clinical and pathological features of MS, the field still lacks a unified mechanistic framework that fully explains disease initiation and progression. The traditional “outside-in” and “inside-out” models alluded to earlier, while conceptually useful, may oversimplify what is likely a far more dynamic and bidirectional process involving continuous interplay between peripheral immunity and CNS-intrinsic factors. Moreover, much of the current understanding is derived from experimental models that do not fully recapitulate the disease heterogeneity observed in humans, particularly across different disease subtypes such as RRMS, SPMS, and PPMS. This heterogeneity extends to clinical presentation, progression rates, and treatment responses, complicating both diagnosis and therapeutic development. Importantly, although neuroinflammation is a central feature across all stages, the relative contribution of inflammatory versus neurodegenerative processes remains unclear and may shift over time. These gaps underscore the need for integrative, systems-level approaches that can better capture the complexity of MS and identify context-specific mechanisms that drive disease variability and progression.

#### 1.2.2. Genetic and Other Risk Factors

MS pathogenesis is multifactorial and involves complex interactions between genetic predispositions and environmental triggers [25]. Over the past several decades, genetic research has been crucial in uncovering pieces of the MS puzzle, offering important insights into the still poorly understood causes of this complex disease. Evidence from both family- and twin-based studies shows that genetic factors play a substantial role in MS susceptibility [26]. For instance, concordance rates are significantly higher in monozygotic (identical) twins (25–30%) than in dizygotic (fraternal) twins (3–7%). This disparity underscores the impact of genetics, while the relatively low concordance among identical twins also points to the disease’s incomplete penetrance—meaning that not everyone with genetic risk factors develops MS [27].

Studies of familial aggregation provide additional evidence for a genetic contribution to MS. The prevalence of the disease among individuals with a family history is estimated to be 15–20%, markedly higher than in the general population [28]. Among first-degree relatives of affected individuals, the lifetime risk is about 3%—approximately 4% for siblings, 2% for parents, and 2% for children. This is around threefold higher than the age-adjusted risk seen in second- and third-degree relatives (about 1%), and roughly 10 to 30 times greater than the risk in the general population, which is estimated at 0.1–0.3% [26,28].

The strongest and most consistently identified genetic association with MS lies within the human leukocyte antigen (*HLA*) gene cluster located on chromosome 6p21 [29]. This 4-megabase region contains approximately 160 closely linked genes. About half of these genes have important roles in the regulation of the immune system which include the six classical transplantation human leukocyte antigen (*HLA*) genes—the class I genes *HLA-A, HLA-B*, and *HLA-C*, and the class II genes *HLA-DPB1*, *HLA-DQB1*, and *HLA-DRB1* [30]). *HLA* genes are highly polymorphic, with over 15,000 alleles identified to date [31].

The *HLA* genes are part of the major histocompatibility complex (MHC), a highly variable genomic region that has long been linked to MS susceptibility. Within this region, associations were initially observed in the class II region known as *HLA-DR2* [32]. This region contains two loci, HLA-DR*15 and HLA-DR*16; however, further genetic analyses refined the MS susceptibility signal to the HLA-DRB1*15 locus [33,34] and more specifically to the HLA-DRB1*15:01 allele [29,33], which are important for the differentiation or function of pathogenic T cells and remain the strongest known genetic risk factors for MS [35,36].

Another genomic susceptibility locus identified for MS is the purine complex upstream of the caveolin 1 gene (*CAV1* gene) [37] that codes for Cav-1 [38]. In fact, genetic studies of Cav-1 have identified several single nucleotide polymorphisms (SNPs) on the *CAV1* gene with potential relevance to human diseases, although direct association with MS remains limited. Among the most studied variants, rs3807989 has been linked to cardiovascular and vascular phenotypes across multiple populations. Additionally, genome-wide and candidate gene studies have associated rs3807989 with susceptibility to atrial fibrillation and coronary artery disease, with some evidence suggesting that the risk allele may influence Cav-1 expression levels and downstream signaling pathways, causing the endothelial dysfunction observed in MS [38]. Whereas the *CAV1* gene is over-expressed in experimental animal models of MS [39,40], there is also increased expression of this gene in Alzheimer’s disease (AD). The loss of this gene, on the other hand, is reported to be associated with neurodegeneration. However, findings remain inconsistent across cohorts, underscoring population-specific effects and genetic heterogeneity, and highlighting the need for further studies to clarify whether these polymorphisms contribute to MS susceptibility or progression. Further discussion of this dichotomy is included in the “Genetic and experimental modes for MS” section.

Although a large Genome-Wide Association Study (GWAS) identified numerous loci associated with MS susceptibility, most of the loci are related to immune function, and not consistently with Cav-1. This suggests that unlike classical MS susceptibility genes, Cav-1 may not be a major driver of disease onset. However, emerging genetic evidence from other neurological disorders provides indirect support for its relevance. For instance, studies in neurodegenerative diseases such as amyotrophic lateral sclerosis have identified rare variants in Cav-1 regulatory regions (e.g., enhancers) that reduce gene expression and disrupt cellular signaling pathways, highlighting its role in neuronal resilience and membrane organization. While these findings are not MS-specific, they suggest plausible mechanisms by which Cav-1 variation could influence neurodegeneration or disease progression rather than susceptibility per se.

Importantly, recent MS genetic research has begun to distinguish between risk-associated variants and those influencing disease progression, with evidence that some variants are linked specifically to disability accumulation rather than disease onset. Within this framework, Cav-1 could be positioned as a modifier gene, potentially affecting processes such as BBB integrity, neuroinflammation, or cellular stress responses. However, direct genetic association studies linking Cav-1 variants to MS progression, severity, or specific phenotypes remain scarce, representing a significant gap in the literature.

Additionally, several limitations in Cav-1 association with MS exist. First, most available studies examining Cav-1 genetics are derived from non-MS conditions, limiting direct extrapolation. Second, even when associations are identified, they often involve non-coding variants with modest effect sizes, making functional interpretation challenging. Third, population heterogeneity and under-representation of diverse cohorts may obscure true associations or lead to inconsistent findings. Finally, the lack of integration between genetic data and functional or clinical outcomes limits the ability to determine whether Cav-1 acts as a causal factor, a modifier, or simply a correlated marker.

Overall, while evidence suggests that Cav-1 may contribute to disease-relevant pathways, the current genetic data do not support a primary susceptibility role in MS. A more balanced interpretation would position Cav-1 as a potential environment-responsive modifier, with its genetic contribution likely subtle and mediated through regulatory rather than coding variation.

There are several other risk factors including environmental and lifestyle risk factors. The most consistent risk factors in MS are childhood obesity, cigarette smoking, and the infection with Epstein–Barr virus (EBV) [22,23,24,41]. On the other hand, high vitamin D levels and sunlight exposure are considered beneficial in MS [25,35]. Polygenic inheritance and dynamic interactions with environmental factors as well as the modest effect sizes of individual variants, combined with influences such as lifestyle and external exposures, make it difficult to disentangle genetic contributions and identify consistent, reproducible risk associations.

#### 1.2.3. Diagnosis

MS diagnosis is based on a combination of characteristic clinical, laboratory, and radiological features integrated into the McDonald criteria [36,37]. These criteria have evolved over time. Specifically, the advent of magnetic resonance imaging (MRI) has made early diagnosis and hence intervention possible [38,41]. Nonetheless, there are still no specific biological markers for MS. IgG oligoclonal bands (OCBs), distinct bands of immunoglobulin G (IgG) antibodies indicating CNS inflammation, are frequently found in MS patients. Thus, cerebrospinal fluid (CSF) analysis plays a pivotal role, as the presence of OCBs in CSF, but not in serum, is crucial for MS diagnosis. When MRI criteria are not met, OCBs can be incorporated into the McDonald criteria as an alternative criterion for dissemination in time (DIT), i.e., evidence that a lesion has occurred at different points in time [37,42]. Visual evoked potentials (VEPs), somatosensory evoked potentials (SSEPs), and optical coherence tomography (OCT) are some of the paraclinical tests in MS that are not yet formally included in the diagnostic criteria [43]. Thus, MS remains a diagnosis of exclusion, making comprehensive evaluation essential for ruling out alternative diseases [37,44].

A critical limitation of the current diagnostic framework is its reliance on indirect and, in some cases, nonspecific indicators of disease activity. While the McDonald criteria have improved sensitivity—particularly with the incorporation of MRI and cerebrospinal fluid findings—this has come with a potential trade-off in specificity, raising concerns about misdiagnosis, especially in atypical presentations of MS. For example, IgG oligoclonal bands, although highly prevalent in MS, are not unique to the disease and may be detected in other inflammatory or infectious conditions of the CNS. Similarly, MRI lesions suggestive of demyelination can overlap with those seen in vascular, metabolic, or other autoimmune disorders. The absence of a definitive, disease-specific biomarker further complicates early and accurate diagnosis, particularly in diverse populations where disease presentation may vary. Moreover, adjunctive tests such as evoked potentials and optical coherence tomography, while informative, lack standardization and formal integration into diagnostic criteria. These limitations underscore the need for more specific biomarkers and refined diagnostic tools that can improve both accuracy and prognostics in MS.

#### 1.2.4. Treatment

Current therapies primarily focus on symptom management rather than on targeting the underlying causes. Thus, future treatment of MS should simultaneously focus on early targeting of peripheral immune cell function and on CNS-intrinsic inflammation, along with potential combination therapy, providing neuroprotection and neuro-regeneration [45]. In this regard, oxidative stress mediators, inflammatory signaling pathways, and the BBB have been widely implicated as molecular targets. However, it is unclear how alterations in molecular substrates influence the development of MS or how they are linked to environmental triggers such as obesity or EBV [2]. A major aim of this review is to elucidate involvement of Cav-1 in dysregulation of these molecular targets particularly their involvement in inflammation and demyelination.

Overall, while the proposed multi-target and integrative therapeutic strategies are conceptually promising, they remain constrained by significant gaps in mechanistic clarity and translational evidence in MS. In particular, the role of Cav-1, although biologically plausible as a central regulator, is not yet sufficiently defined to support its prioritization as a therapeutic target. Addressing this limitation will require more rigorous, context-specific studies that distinguish causation from association and clarify how Cav-1 interfaces with immune, neurovascular, and environmental factors across disease stages. Until such evidence is available, its clinical utility should be considered exploratory rather than definitive.

#### 1.2.5. Genetic Models of MS

MS naturally occurs only in humans; however, different animal models have been developed to mimic MS. The current models can be grouped into three categories based on the nature of agents used to induce the condition: autoimmune, viral, and neurotoxic [46]. Experimental autoimmune encephalomyelitis (EAE) is the oldest and most frequently used model system for studying MS in laboratory animals and falls in the first category. Rather than a single model, EAE is a family of models in which CNS inflammation occurs after immunization against CNS-specific antigen [47]. In its classic form, EAE is a CD4+ T-cell-mediated autoimmune disease in which immunization with myelin proteins or peptides induces the migration of activated autoreactive T cells across the BBB and into the CNS. Similarly, transfer of autoreactive T cells activated by such antigens can achieve the same result [48]. Indeed, transgenic humanized mice expressing MS-associated HLA (e.g., HLA-DR2) and human myelin-specific T-cell receptors (TCRs) (e.g., MBP84-102-specific) develop EAE and provide a crucial model for studying MS [49]. Interestingly, Cav-1-dependent neuroinflammatory chemokine CXCL10 promotes CD4+ T cell transmigration across brain endothelial cells [50], suggesting a role for Cav-1 in autoimmune disorders such as MS.

Like MS, the *MHC* locus displays the biggest contribution to EAE susceptibility and manifestation, confirming the important role of T cells and antigen presentation in disease pathogenesis [51]. In addition, at least 27 non-*MHC* loci (*Eae1*-*Eae27*) have been found to be associated with different traits of the disease, including its incidence, onset, severity, and histopathology [52,53,54]. Interestingly, many of these loci show sex specificity, possibly underscoring gender susceptibility in MS. Moreover, low levels of transducer of ERBB.2-1 (*TOB1*) transcript in CD4+ T cells are strongly associated with a higher risk of early conversion to clinically defined MS in patients experiencing a first demyelinating event in the CNS [52,55].

The *CAV1* gene is over-expressed in experimental animal models of MS [39,40]. The wild-type mice with active encephalomyelitis also show increased expression of Cav-1 in serum and spinal cord tissues in parallel with disease incidence and severity [39,40,56]. On the other hand, Cav-1 knockout mice show lower encephalitogenic T cells trafficking into the CNS and reduced expressions of adhesion molecules ICAM-1 and VCAM-1 within the lesions [57]. These mice also demonstrate remarkable disease resistance after immunization [39,40]. This suggests that Cav-1 knockdown limited the upregulation of ICAM-1 in endothelial cells, leading to the reduction of the trans-endothelial migration of pathogenic T-helper 1 (Th1) and T-helper 17 (Th17) cells [57]. Involvement of ICAM-1 in neurodegenerative diseases has been recently reviewed [58]. In this regard, it has been suggested that abnormal levels of Cav-1 and ICAM-1 in serum and CSF may serve as markers for active MS, reflecting BBB damage, neuroinflammation, and brain lesions underscored by demyelination [59,60,61,62].

While experimental models have provided valuable insights into the immunopathogenesis of MS, their translational relevance remains limited. EAE predominantly models a CD4+ T-cell-driven autoimmune process, which captures important aspects of inflammation and BBB dysfunction but does not fully reflect the complexity, heterogeneity, and progressive neurodegenerative features observed in human MS. Similarly, genetic associations identified in EAE, including MHC and non-MHC loci, may not directly translate to human disease due to species differences and controlled experimental conditions. The observed involvement of Cav-1 in these models—such as its role in T cell trafficking, endothelial activation, and disease severity—provides compelling mechanistic evidence; however, these findings are largely derived from induced systems that may exaggerate specific pathways. Moreover, the apparent protective effect seen in Cav-1 knockout mice highlights its potential as a therapeutic target but also underscores the risk of oversimplification, given its roles across different cell types. The proposal of Cav-1 and ICAM-1 as biomarkers is intriguing, yet remains preliminary, with limited validation in human cohorts. Overall, while these models are indispensable for mechanistic exploration, caution is warranted in extrapolating their findings to clinical MS, emphasizing the need for human-based studies to confirm the role of Cav-1 in disease pathogenesis and progression.

### 1.3. Molecular-Genetic Substrates of MS

#### 1.3.1. Oxidative Stress—MS

Under physiological conditions, reactive oxygen and nitrogen species (ROS/RNS) play pivotal roles in the homeostasis of the cellular environment; however, in pathological conditions, they are involved with the initiation and the perpetuation of the inflammatory and neurodegenerative processes. Additionally, both central and peripheral mechanisms, via enzymes such as NADPH oxidase (NOX), xanthine oxidase (XO), nitric oxide synthase (NOS), myeloperoxidase (MPO), and catalase (CAT) induce oxidative stress [63,64]. On the other hand, enzymes such as superoxide dismutase (SOD), catalase, or glutathione peroxidase (GSH-Px) function as antioxidants [65,66]. Of note, the body’s primary defense against oxidative stress involves the Nrf2-Keap1-ARE pathway [67].

Oxidative species are highly reactive and have the capacity to degrade proteins, lipids, and DNA [66,68], thereby compromising cellular function and integrity [69,70]. Moreover, accumulation of ROS in the brain can cause neuronal injury and cell death due to neuroinflammation [71,72]. Oxidative stress also disrupts the integrity of the BBB, resulting in increased permeability and functional impairment. It is noteworthy that both neuroinflammation and BBB dysfunction are key features in the progression of neurological disorders such as MS [66]. This disruption allows inflammatory cells and potentially harmful substances to infiltrate the brain, further contributing to neuronal damage and the progression of neurodegenerative diseases [73]. Neuropathic and inflammatory pain are also associated with elevated levels of ROS [74,75].

Whereas the systemic or intrathecal administration of ROS scavengers and antioxidants inhibit the pain behavior in various animal models of neuropathic pain [76], tobacco consumption increases the risk of developing MS via activation of the above-mentioned mechanisms [77,78]. Nonetheless, oxidative stress and inflammatory processes are believed to be major contributors to the tissue damage observed in MS [79,80]. This contention is further supported by observations in the EAE model where elevated levels of ROS produced by macrophages can damage myelin and axons [81]. However, the role of specific genes and their impact on the disease remain to be elucidated. Interestingly, a potential link between the oxidative stress-responsive *STAT3* gene and MS has been suggested. This is because activation of this gene transforms macrophages from a detrimental M1 phenotype to a beneficial M2 phenotype [82,83]. Moreover, STAT3 plays a crucial role in myeloid cell activation, T-cell polarization, and cytokine/chemokine production, all of which are involved in MS [84,85]. Interestingly, oxidative stress affects Cav-1 expression and function, which in turn alters endothelial stability and inflammatory responses.

While oxidative stress is clearly implicated in the pathophysiology of MS, it is often described in broad and associative terms rather than within a well-defined causal framework. Although increased ROS/RNS are consistently linked to neuroinflammation, BBB disruption, and neuronal injury, it remains unclear whether oxidative stress acts as a primary driver of disease or as a secondary consequence of ongoing inflammation. Much of the supporting evidence derives from experimental models or from indirect clinical observations, which may not fully capture the temporal and spatial complexity of oxidative processes in human MS. Furthermore, antioxidant-based therapeutic strategies, despite strong preclinical rationale, have shown limited and inconsistent efficacy in clinical settings, raising questions about the translatability of targeting oxidative stress alone. The involvement of signaling pathways such as STAT3 and regulators like Cav-1 highlights additional layers of complexity, as these molecules exert effects on immune activation, endothelial function, and cellular survival. However, the precise interactions between oxidative stress, gene regulation, and Cav-1-mediated signaling remain insufficiently characterized. Overall, while oxidative stress is undoubtedly a key component of MS pathology, its exact mechanistic role and therapeutic exploitability require more precise, integrative investigation.

#### 1.3.2. Endothelial Cell—BBB—MS

Microvessels’ endothelial cells of the CNS tightly regulate the movement of ions and molecules as well as immune cell trafficking into the CNS by forming the BBB. The BBB is crucial for maintenance of a homeostatic environment. Damage to brain endothelial cells leads to an influx of deleterious molecules into the CNS, accelerating leakage across the BBB, where leukocyte entry into the CNS marks an early event in MS. Moreover, endothelial capabilities in antigen presentation and immune cell recruitment directly initiate and amplify neuroimmune responses [86,87]. However, whether BBB dysfunction precedes immune cell infiltration or is the consequence of perivascular leukocyte accumulation remains unknown, but leukocyte migration modifies BBB permeability. In the early stages of MS, around the time of symptom onset, inflammatory BBB damage is accompanied by pathogenic immune cell infiltration into the CNS [88]. In the later stages of MS, dysregulation of neurovascular coupling is believed to lead to gray matter atrophy [88].

Nonetheless, BBB disruption is widely recognized as an early and defining feature of MS. It remains unclear whether this disruption represents a primary initiating event or a secondary consequence of immune activation and leukocyte trafficking. Much of the current evidence is derived from imaging studies and experimental models, which may not fully capture the dynamic and potentially reversible nature of BBB alterations in human disease. Furthermore, the role of endothelial cells as active participants in antigen presentation and immune modulation adds complexity, suggesting that the BBB is not merely a passive barrier but an immunologically active interface. However, this dual role complicates therapeutic targeting, as interventions aimed at stabilizing the BBB may inadvertently affect essential immune surveillance or CNS homeostasis. Additionally, BBB dysfunction is heterogeneous across disease stages and regions of the CNS, and its contribution to later neurodegenerative processes—such as gray matter atrophy—remains incompletely defined. These uncertainties highlight the need for more precise temporal and mechanistic studies to determine how BBB alterations integrate with immune and neurodegenerative pathways in MS.

#### 1.3.3. Inflammation—MS

T and B cells are key components of the adaptive immune system. Through their immune properties and their interactions with other immune cells and cytokines, they build a complex network to achieve immune tolerance and maintain homeostasis throughout the body [89]. The immune system must maintain a finely tuned balance between mounting effective defenses against external insults and avoiding harmful responses to self-antigens. This critical equilibrium is achieved through immune tolerance and long-term constraint of potentially harmful responses towards innocuous stimuli [89,90]. For this reason, dysregulation in this tightly regulated system can result in various diseases including autoimmune disorders such as MS. Thus, a breakdown of immune tolerance within the CNS, despite its semi-independence from the peripheral immune system, can lead to MS [91,92,93,94].

Notably, myeloid cells are also important contributors to MS pathology. In this regard, it is suggested that pathogenesis of MS starts with the escape of autoreactive T cells from clonal deletion in the thymus followed by their reactivation in lymphoid tissues and their crossing into the CNS, causing gliosis, oligodendrocytes damage, and demyelination [59,95]. The late stages of MS are accompanied by compartmentalized inflammation, contributing to continuous inflammatory and degenerative changes in the CNS, hence driving disease progression [60,61,96,97]. The pathogenesis of MS is mainly mediated by CD4+ T cells, which recognize myelin-like peptides in the periphery and then infiltrate the CNS, where they trigger an immune attack toward destruction of myelin [62,63,64,98,99,100].

During the course of the disease, the infiltration of Th1 and Th17 autoreactive effector T cells causes an increase in pro-inflammatory cytokines such as interferon-γ (IFN-γ) or TNFα [22]. This induces excessive production of ROS and RNS which further promote inflammation and cause oxidative stress [17,101]. The Th17 cell and regulatory T cell (Treg) axis plays a crucial role in the development of MS, which is regarded as an immune imbalance between pro-inflammatory cytokines and the maintenance of immune tolerance [102]. Myelin autoreactive CD4^+^ T cells from MS patients present higher antigen avidity and show a skewed pro-inflammatory profile compared to healthy controls [103]. However, efficient trafficking and extravasations of these highly pathogenic immune cells into the CNS are prerequisites for triggering neuroinflammation and MS development [104].

A critical limitation of this immunological framework is that while T and B cells—particularly autoreactive CD4+ subsets such as Th1 and Th17—are clearly central to the pathogenesis of MS, this perspective may oversimplify the broader and more dynamic immune landscape. Much of the current model is heavily informed by experimental systems, which emphasize CD4+ T-cell-driven mechanisms but may not fully capture the contributions of other immune populations, including B cells, myeloid cells, and CNS-resident microglia, especially in progressive stages of the disease. Furthermore, the concept of immune tolerance breakdown, although widely accepted, does not fully explain why autoreactive cells escape regulation in some individuals but not others, nor how peripheral immune events are coordinated with CNS-specific triggers. The Th17/Treg imbalance provides a useful framework, yet it remains unclear whether this imbalance is a primary cause or a downstream effect of ongoing inflammation. In addition, the requirement for immune cell trafficking into the CNS highlights the importance of neurovascular interfaces, but the precise signals governing this process are incompletely understood. Collectively, these gaps underscore that MS cannot be fully explained by a single immune axis; rather, it reflects a complex interplay between adaptive immunity, innate responses, and CNS-intrinsic factors that evolve across disease stages.

#### 1.3.4. Autophagy—M

Autophagy, derived from Greek to depict “self-eating,” is an essential cell-recycling process that, by removing damaged cellular components, provides opportunity for cellular repair or rejuvenation. It occurs within lysosomes and is critical for maintaining the immune function as well as the overall health of the cell [105,106]. In this context it is a major player in induction and management of chronic inflammatory diseases [106,107].

However, in MS, autophagy may play a dual role. On one hand, it enhances remyelination by increasing the activity of oligodendrocytes, and astrocytes, and on the other hand, by activating microglia and T cells, it may induce demyelination. Nonetheless, targeted modulation of autophagy to reduce neuroinflammation and promote repair is being explored for MS intervention [108]. In this regard, Cav-1 regulates autophagic processes that feed back into redox balance and immune signaling.

In MS, inflammation, BBB dysfunction, oxidative stress, and autophagy are tightly interconnected processes that form a self-reinforcing network rather than independent pathways. Pro-inflammatory cytokines released by activated immune cells disrupt BBB integrity by altering tight junctions, increasing permeability and enabling further leukocyte infiltration into the CNS. This amplified immune cell entry intensifies local inflammation and promotes the generation of reactive oxygen species, leading to oxidative stress and mitochondrial dysfunction. Elevated oxidative stress, in turn, impairs autophagic flux, disrupting cellular homeostasis and reducing the ability of cells to clear damaged proteins and organelles. Dysfunctional autophagy further exacerbates inflammatory signaling and cellular injury, thereby feeding back into the inflammatory cascade. Together, these processes create a dynamic and interdependent loop in which each pathway both influences and is influenced by the others, driving disease progression and tissue damage in MS.

### 1.4. Cytoskeleton and Cell Membrane Interaction

The cytoskeletal elements within the cell’s cytoplasm are formed from a complex and dynamic network of protein filaments that provide internal scaffolds and structural support that not only maintain the cell’s shape but also participate in cellular activities. The cytoskeleton comprises three main types of protein fibers: microfilaments (actin-based, involved in movement), intermediate filaments (mechanical strength), and microtubules (tubulin-based, transport tracks) [109]. Each of the components displays a highly organized structure contributing to multifaceted functions [110]. All three proteins are capable of rapid assembly and disassembly, allowing the cell to quickly adapt its internal architecture [109]. Most of the interactions between the cytoskeleton and the cell membrane appear to involve actin [111].

The outer boundary of every cell, the cell membrane, helps transport molecules and ions and allows cell-to-cell communication. This communication with the extracellular environment heavily depends on interactions between cell membrane organelles and cytoskeletal elements as well as continuous remodeling of the cell structure [112]. While the cell membrane provides docking sites for cytoskeletal elements and serves as the source of the signaling molecules that control cytoskeletal organization and remolding [113], the cytoskeleton, on the other hand, determines the biophysical and biochemical properties of the membrane. Thus, the cell membrane–cytoskeleton interplay underlies—as a central regulator—a multitude of complex processes including signal transduction, motility/migration, membrane traffic, cell–cell, and cell–matrix adhesion [113]. These events in turn impact development and tissue differentiation. Thus, cell membrane–cytoskeleton interactions are central to deciphering how cytoskeletal remodeling, organelle transport, and cytoskeletal organization are integrated [114]. When the intricate relationship between the cell membrane and the cytoskeleton is disrupted, significant consequences on cellular health become evident.

### 1.5. Caveolae and Caveolins

Caveolae (Latin for “little caves”) are 60–80 nm wide flask-shaped invaginated pits on the cytoplasmic membrane that can bud to generate endocytic vesicles and uptake pathogens and compartmentalize certain signaling molecules, hence, significantly affecting the efficiency of the cell’s signal transduction [115]. It is noteworthy that compared to the endothelium of non-neural vessels, CNS endothelial cells have limited vesicular transports (transcytosis) that are up to 14-fold fewer. Thus, numerous macromolecules, including albumin, lipoproteins, insulin, and transferrin, are trans-endothelially delivered through caveolae [116].

#### 1.5.1. Cav-1

Caveolin (Cav) is an integral structural protein forming caveolae [117]. There are three subtypes of caveolins: caveolin-1 (Cav-1), caveolin-2 (Cav-2), and caveolin-3 (Cav-3). Cav-1 and Cav-2 are widely expressed in fibroblasts, adipocytes, neuronal cells, and endothelial/epithelial cells whereas Cav-3 is muscle specific. Of these, Cav-1 is essential for caveolae formation. Each caveola consists of 140–150 Cav-1 molecules. The core component of Cav-1 is co-expressed with cavin1 (Cav-1 adaptor protein) to form caveolae [118]. Cav-1 is the most extensively studied and has functions independent of caveolae [118,119,120]. The abnormal expression of Cav-1 and the dysfunction of caveolae are closely related to various diseases, including neurological diseases [121]. Thus, our focus will be on Cav-1.

Cav-1 has two isoforms: Cav-1α (24 kDa) and Cav-1β (21 kDa). Both isoforms have a complete C-terminal. However, Cav-1β lacks the N-terminal-specific protein sequences (residues 1–21) [122]. Once synthesized and oligomerized, Cav-1 is inserted into the endoplasmic reticulum (ER) membrane. The hydrophobic domain serves as an ER membrane anchor while the N-terminal domain allows Cav-1 to attach to the exit sites of ER that can then be transported to the Golgi apparatus [123]. In the Golgi apparatus, Cav-1 is assembled into larger and more stable complexes of about 160 caveolin molecules, containing lipids and membrane raft-associated cargos [88]. These are then transported to the plasma membrane as vesicles and inserted as planar caveolar domains to generate the caveolae structures [124].

Cav-1 in caveolae is tethered to cortical actin via direct interaction with the actin-binding protein filamin, stabilizing caveolae on the plasma membrane and simultaneously coupling to the cytoskeleton [125]. Because these proteins can be internalized, Cav-1 is also detectable at many intracellular sites [126,127]. Although their precise role in intracellular sites remains obscure, it is suggested that they play a role in epidermal growth factor receptor (EGFR) signaling, endocytosis, and focal adhesion dynamics [128]. Cav-1 phosphorylation is required for its interaction with other proteins, clustering of specific lipids, and overall function, which also involves morphological changes in caveolae [129,130].

Cav-1 is essential for multiple biological processes including membrane trafficking, cholesterol homeostasis, endocytosis, receptor internalization, cell proliferation, cell death, and cell signaling [131,132]. In fact, Cav-1 facilitates diverse signaling pathways with numerous interacting partners, including the EGFR and endothelial NOS [133]. Moreover, Cav-1 recruits and organizes several signaling proteins, including receptors, kinases, and G proteins, thereby tuning the strength and duration of inflammatory signals [132,134]. Cav-1 is also involved in calcium signaling, autophagy, and apoptosis [135]. Cav-1 should be understood not as having a singular role, but as a dynamic regulator whose effects depend on disease stage, cellular context, and regulatory balance.

#### 1.5.2. Cav-1—Inflammation—Oxidative Stress—MS

While Cav-1 is a structural component of myelin, its presence in inflammatory settings promotes the trafficking of immune cells that cause demyelination [37]. Thus, Cav-1 is implicated in inflammation initiation, and neurodegeneration [136,137,138]. However, Cav-1, as an environment-responsive regulator of inflammation, sometimes promotes and other times restrains inflammatory responses depending on the cell type, stimulus, and disease setting. Overall, Cav-1 facilitates inflammatory signaling by organizing receptors in caveolae, modulating innate immune pathways, and coupling these to autophagy, endothelial activation, and cytokine production. For example, in wild-type mice with active EAE, increased expression of Cav-1 in serum and spinal cord astrocyte populations is associated with disease incidence and severity [56,57,139]. On the other hand, lack of endothelial Cav-1 reduces the infiltration of Th1 cells into the CNS, resulting in decreased overall neuroinflammation and severity of EAE [140]. However, how Cav-1 interacts with cytoskeletons, and its role in inflammation associated in MS, is yet to be fully elucidated [141,142]. Moreover, it is essential to consider the heterogeneity of Cav-1 regarding its beneficial vs. harmful effects [139].

Bruton tyrosine kinase (BTK) is a Tec family tyrosine kinase that is crucial for B cell development, differentiation, and signaling [143]. Thus, pharmacologic inhibition of BTK prevents activation of downstream signaling pathways preventing antigen-driven activation and proliferation as well as B-cell-dependent T-cell activation. In this regard, Cav-1 emerges as a cell-intrinsic regulator that prevents B-cell-induced autoimmunity via plasma-membrane organization [144]. Moreover, Cav-1 downregulates tyrosine phosphorylation of BTK [145]. Although the functional significance of this interaction is not presently understood, the negative regulation of BTK activity by Cav-1 may represent a relevant consequence of the different signaling pathways where BTK is involved. Among the therapies currently being investigated for use in progressive MS are BTK inhibitors such as Fenebrutinib.

As alluded to earlier, the body’s primary defense against oxidative stress involves the Nrf2-Keap1-ARE pathway [67]. It is noteworthy that Cav-1 constitutively interacts with Nrf2 in both the cytosol and nucleus [146]. Interestingly, smoking may increase the risk of MS by decreasing factors related to antioxidant capacity, such as uric acid, as well as upregulating Cav-1 [6,147]. Curiously, loss of Cav-1 also increases ROS production and may contribute to fibrosis [6,148]. Thus, a thorough understanding of Cav-1 interaction with the Nrf2-Keap1-ARE pathway may lead to the development of novel treatments for a broad range of human diseases including MS.

Collectively, current evidence suggests that Cav-1 occupies a central position at the intersection of inflammation and oxidative stress, two key processes underlying tissue damage in MS. While Cav-1 appears to modulate redox signaling, immune activation, and endothelial function, its precise role remains complex, with both protective and detrimental effects reported depending on cellular context and disease stage. Importantly, the lack of consistent genetic and mechanistic evidence in MS highlights a critical gap in understanding this complex neurological disorder. Thus, integrative, large-scale, and longitudinal studies are required to clarify whether Cav-1 represents a viable therapeutic target or simply an environment-responsive mediator within the broader pathogenic networks.

#### 1.5.3. Cav-1—Myelination—MS

In myelinated nerves, Cav-1 is localized in the outer myelin membranes, where it acts as a scaffold for signaling proteins and cholesterol trafficking necessary for maintaining myelin integrity [116,149]. In this context, changes in Cav-1 regulation and protein rearrangements can induce immune response and inflammation [150,151]. Although some studies suggest that Cav-1 is associated with promoting inflammation in certain conditions, it may also act as an inhibitor of eNOS activity [152]. Since excessive eNOS may contribute to vascular leakiness, Cav-1 or cavtratin, derived from scaffolding domain peptides, can stabilize the BBB and reduce pathological permeability [153]. Cav-1 is also required for the expansion of the oligodendrocyte membrane, a process essential for wrapping axons during development [154]. Here also, Cav-1 is significantly upregulated during the maturation of both oligodendrocytes and Schwann cells [155].

Recent findings highlight “oligovascular coupling,” where endothelial Cav-1 stabilizes the interaction between blood vessels and oligodendrocyte precursor cells (OPCs). Maintaining this coupling is essential for successful oligodendrogenesis and demyelination prevention [156]. However, elevated levels of Cav-1 in neural progenitor cells (NPCs) and OPCs may inhibit their differentiation into mature, myelin-producing oligodendrocytes [157]. This is likely due to suppression of the Wnt/β-catenin pathway, which is critical for oligodendrocyte development [158]. Moreover, excess Cav-1 can suppress β-catenin, thereby stalling the transition from progenitor to mature oligodendrocyte [159]. Thus, maintaining a balanced Cav-1 appears to be critical in myelination and overall cellular function.

Thus, Cav-1, on one hand, supports myelin integrity, BBB stability, and oligodendrocyte–vascular coupling, but on the other hand, excessive or dysregulated Cav-1 can impair progenitor cell differentiation—likely through modulation of the Wnt/β-catenin signaling pathway—thereby hindering remyelination. This dual role underscores that optimal Cav-1 homeostasis, rather than simple upregulation or inhibition, is critical for maintaining cellular balance and may be particularly relevant to the pathophysiology of MS.

#### 1.5.4. Cav-1—BBB—MS

Cav-1 modulates inflammatory signaling pathways that directly influence BBB permeability and leukocyte trafficking. Interestingly, elevated levels of Cav-1 promote endothelial cell transcytosis and the internalization of tight junction proteins like occludin and claudin-5 which break down the protective barrier, allowing massive infiltration of inflammatory cells that can exacerbate MS symptoms [159]. Genetic and environmental factors associated with MS, such as dietary habits, gut microbiome, and vitamin D levels, might contribute directly or indirectly to brain endothelial cell dysfunction. Cav-1 can induce endothelial cell dysfunction through a variety of mechanisms including decreasing protective autophagy in endothelial cells [138]. Moreover, in experimental models of cerebral ischemia, downregulation of Cav-1 results in increased BBB permeability [160]. Curiously, in EAE, suppression of Cav-1 by Cav-1 scaffolding domain peptides reduces inflammatory cell infiltration and improves BBB function [139]. Thus, therapeutic approaches targeting Cav-1 may not only prevent BBB damage but also help its repair. However, in this case also it appears that an optimal level of Cav-1 is the desired endpoint as both excess and deficiency can have detrimental effects.

#### 1.5.5. Cav-1—Autophagy—MS

While several intracellular signaling pathways and multiple autophagy-related proteins regulate autophagy, Cav-1 has emerged as an autophagy modulator. Thus, under oxidative stress, Cav-1 binds with activated BECN1 (autophagy protein)/VPS34 (lipid kinase activity of PI3K-III catalytic unit) complex and translocates into mitochondria, where it further induces autophagy [161,162,163]. However, the role of Cav-1 might be condition-dependent as it is not necessarily a prerequisite for autophagy activation [163]. On the other hand, autophagy itself may modulate (degrade) Cav-1 to induce cell apoptosis and inflammation [164], which complicates a clear conclusion on the relationship between Cav-1 and autophagy.

The picture becomes more complex as Cav-1 deficiency has also been associated with autophagy promotion and protective effects against diseases. For example, Cav-1 knockout mice show reduced inflammation and pain [165]. However, there is controversy on this point as well, as other studies have shown that Cav-1 loss is associated with increased IL6 and TNFα production in several inflammatory and autoimmune settings [166]. Moreover, Cav-1 knockout mice exhibited a low grade systemic pro-inflammatory state, with elevated circulating IL1β, IL2, IL6, IL12, and TNFα [167]. Similarly, downregulating Cav-1 expression in murine macrophages increased LPS-induced pro-inflammatory cytokine TNF-α and IL-6 production and decreased the production of the anti-inflammatory cytokine IL-10. Conversely, overexpression of Cav-1 in the mouse macrophage cell line RAW264.7 decreased LPS-induced TNF-α and IL-6 production and augmented IL-10 production [168].

These results show that Cav-1 involvement in cell homeostasis vis-a-vis autophagy is more complex than previously thought. Nonetheless, it is suggested that further elucidation of this interaction could be of therapeutic benefit [165,169].

Overall, the evidence indicates that Cav-1 is a context-dependent regulator of autophagy and inflammatory homeostasis rather than a unidirectional modulator. Although under oxidative stress Cav-1 can interact with the BECN1/VPS34 complex to promote autophagic responses, it is not strictly required for autophagy initiation and may itself be subject to autophagic degradation, linking it to downstream apoptotic and inflammatory outcomes.

Moreover, conflicting findings from Cav-1 deficiency models—ranging from reduced inflammatory phenotypes to heightened pro-inflammatory cytokine production—highlight its dual and context-specific roles in immune regulation. This suggests that Cav-1 is unlikely to be purely beneficial or harmful; rather, it may act as a disease modifier, with its effects varying depending on disease stage, cell type, and microenvironment. For example, early in the disease, Cav-1 might help preserve barrier function, whereas in later stages it could amplify inflammatory cascades. Therefore, Cav-1 is best interpreted not simply as a marker, but as a dynamic regulator that can both mitigate and exacerbate disease processes in MS. Collectively, these inconsistencies underscore a highly dynamic Cav-1–autophagy–inflammation axis, suggesting that therapeutic targeting will require precise contextual and cell-type-specific understanding, particularly in complex diseases such as MS.

### 1.6. Cav-1 and CAV1 Gene—MS

Mice deficient in the *CAV1* gene exhibit a variety of conditions, including cardiovascular disease, diabetes, cancer, atherosclerosis, and pulmonary fibrosis [137,138,170,171,172,173]. Although the underlying mechanisms responsible for these diverse phenotypes are not yet fully understood, it has been suggested that the absence of Cav-1 may interfere with metabolic regulation. For instance, Cav-1−/− mice display impaired liver regeneration unless supplemented with glucose, pointing to systemic metabolic deficiencies that require additional intermediates [139,174,175]. Establishing a direct functional link between Cav-1 and metabolic processes could help provide a unifying explanation for these varied disease manifestations [176,177,178].

Thus, the future treatment of MS should simultaneously focus on early intervention, potentially using Cav-1 and soluble ICAM-1 as biomarkers, targeting peripheral immune cell function and CNS-intrinsic inflammation, along with potential combination therapy, providing neuro-regeneration and neuroprotection [43]. This is particularly critical for MS as pathological mechanisms remain unclear and there is no biomarker currently available. Elevated levels of soluble ICAM-1 in the blood are markers of active disease and new brain lesions in MS patients [179]. Secreted Cav-1 levels in serum may serve as a potential early biomarker to index the clinical severity of MS, as serum levels correlate with disease progression in preclinical models [139].

In short, Cav-1 expression underscored by the *CAV1* gene may prove a reliable biomarker in MS, as its expression correlates with the disease severity and progression. Moreover, its role in regulation of the BBB, glial cell dysfunction, and immune cell infiltration into the CNS, leading to neuroinflammation, makes it a suitable candidate for therapeutic intervention as well [169,179].

**Figure 1:**
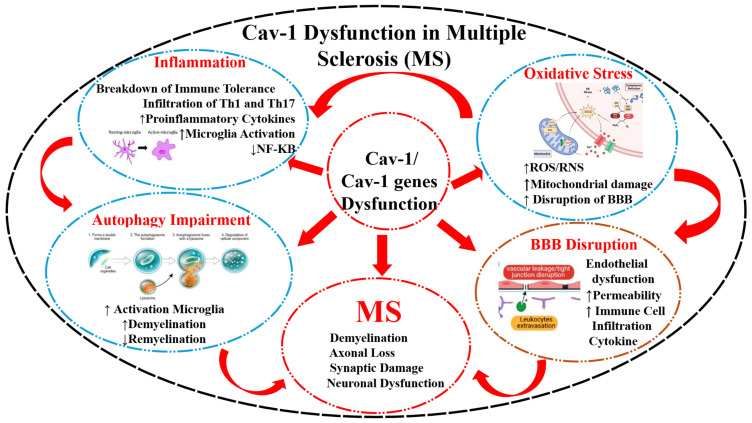
Schematic diagram showing how dysfunction in Cav-1 genes or protein expression may affect the immune system as well as the blood–brain barrier (BBB) and lead to demyelination and multiple sclerosis (MS). Dysregulation of Cav-1/Cav-1–related genes is proposed to contribute to multiple pathological mechanisms involved in MS. **Solid red arrows** indicate direct pathogenic effects or signaling interactions originating from Cav-1 dysfunction toward downstream processes, including inflammation, oxidative stress, BBB disruption, autophagy impairment, and the development of MS pathology. **Curved red arrows** represent positive feedback loops and bidirectional amplification among these pathological pathways, indicating that each process can further exacerbate Cav-1 dysfunction and disease progression. Cav-1 dysfunction promotes inflammatory responses characterized by breakdown of immune tolerance, infiltration of Th1/Th17 cells, increased pro-inflammatory cytokines, microglial activation, and altered NF-κB signaling. It also contributes to oxidative stress through increased reactive oxygen/nitrogen species (ROS/RNS), mitochondrial damage, and BBB injury. BBB disruption leads to endothelial dysfunction, increased vascular permeability, immune cell infiltration, and cytokine release. Impaired autophagy enhances microglial activation, demyelination, and defective remyelination. Collectively, these interconnected mechanisms culminate in hallmark MS pathology, including demyelination, axonal loss, synaptic damage, and neuronal dysfunction.

**Figure 2:**
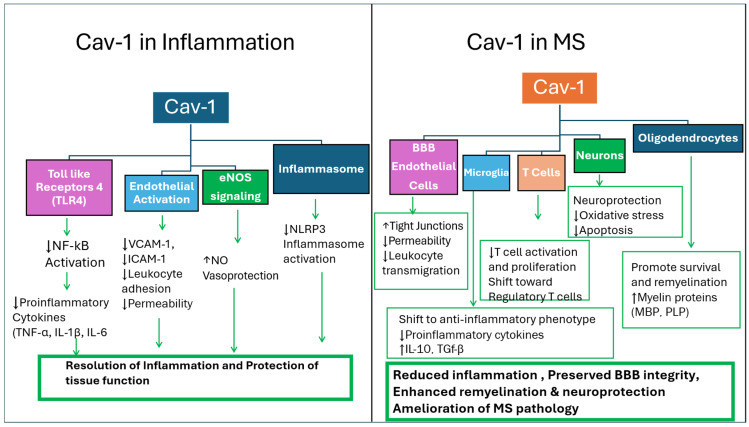
Cav-1 regulates inflammatory signaling and modulates MS pathology. This schematic illustrates the role of Cav-1 as a membrane scaffolding protein that organizes signaling complexes within caveolae and regulates inflammatory pathways. The left panel illustrates the anti-inflammatory and vasoprotective functions of Cav-1 in general inflammatory conditions. Cav-1 modulates multiple signaling pathways, including Toll-like receptor 4 (TLR4), endothelial activation, endothelial nitric oxide synthase (eNOS) signaling, and inflammasome activation. Through these pathways, Cav-1 suppresses NF-κB activation, reduces pro-inflammatory cytokine production (TNF-α, IL-1β, and IL-6), decreases endothelial adhesion molecules (VCAM-1 and ICAM-1), limits leukocyte adhesion and vascular permeability, enhances nitric oxide (NO)-mediated vasoprotection, and inhibits NLRP3 inflammasome activation, collectively contributing to the resolution of inflammation and preservation of tissue function.

Thus, Cav-1 may have translational value as a biomarker in MS, rather than as a standalone or disease-specific indicator. This context dependence means that Cav-1 should not be interpreted in isolation, but rather as part of a broader panel of markers capturing different aspects of disease activity. Its utility may lie in complementing existing diagnostic and monitoring tools, providing additional insight into the neurovascular and inflammatory dynamics that are not fully captured by current approaches. However, variability across cell types, disease stages, and experimental systems remains a key limitation, underscoring the need for standardized measurement strategies and validation in well-characterized patient cohorts. These mechanisms are summarized in Figure 1 and Figure 2 as well as in Table 1.

### 1.7. Therapeutic Potential

Specific strategies for modulation of Cav-1 may include upregulation of its expression in endothelial cells to stabilize the BBB, or conversely, to inhibit Cav-1-mediated signaling in immune cells if it is shown to promote inflammation. Pharmacological modulation (e.g., small molecules, peptides targeting caveolae-associated pathways), gene regulation strategies, or cell-specific delivery systems could all be considered. However, the feasibility of these approaches depends heavily on achieving cell-type specificity, as global modulation of Cav-1 may produce conflicting or unintended effects.

Synthetic peptides derived from the Cav-1 scaffolding domain have been used experimentally to stabilize endothelial signaling and reduce permeability of the BBB. In MS and its animal model, EAE, BBB disruption is a key early event. Enhancing Cav-1 function via Cav-1 scaffolding domain (CSD) peptides, for example, can suppress inflammatory signaling pathways, reduce leukocyte infiltration into the CNS, and improve barrier integrity—suggesting a strategy to limit neuroinflammation. Moreover, Cav-1 influences multiple signaling pathways involved in inflammation (e.g., NF-κB, cytokine production). In EAE models altering Cav-1 expression can change disease severity: increasing Cav-1 activity in some contexts reduces pro-inflammatory cytokines and immune cell activation, while its dysregulation is associated with enhanced inflammation and demyelination. Targeting Cav-1-dependent signaling pathways therefore offers a potential strategy for modulating immune responses in MS.

A major limitation that should be explicitly addressed is the environment-responsive role of Cav-1, which complicates its therapeutic targeting. Cav-1 has been reported to exhibit both protective and detrimental functions depending on the cellular environment and disease stage. For example, in endothelial cells, Cav-1 may help maintain BBB integrity and limit leukocyte infiltration, suggesting a protective role. In contrast, in immune cells, Cav-1 may facilitate inflammatory signaling pathways and enhance immune activation, potentially worsening disease pathology. This duality raises an important challenge: interventions that increase Cav-1 activity could be beneficial in one compartment but harmful in another.

The literature also contains apparent contradictions, particularly regarding the pro- versus anti-inflammatory roles of Cav-1. These opposing findings may reflect differences in experimental models, disease stages, or cell-specific effects. Thus, Cav-1 is a dynamic regulator whose net effect depends on the balance of these factors.

Overall, while Cav-1 represents a promising target, its therapeutic potential remains conditional. The success of Cav-1–based interventions will likely depend on precise targeting, timing, and an improved understanding of its roles.

## 2. Conclusions

In conclusion, within the complex and evolving landscape of MS, Cav-1 is most appropriately viewed not as a primary driver of pathology, but as a context-dependent regulatory node that integrates signaling across endothelial, immune, and neural compartments. As a membrane-associated scaffold protein, Cav-1 modulates multiple interconnected pathways, and its functional impact is highly contingent on cellular context, disease stage, and microenvironmental conditions. Under physiological or early disease states, Cav-1 may exert protective effects, including maintenance of BBB integrity and regulation of controlled immune responses. In contrast, within pro-inflammatory or oxidative environments, it may adopt detrimental roles, promoting leukocyte trafficking, amplifying inflammatory signaling, and contributing to cellular dysfunction. This dual and dynamic behavior underscores its role as a modifier of disease processes, capable of shaping their magnitude and trajectory rather than initiating them.

From a translational perspective, Cav-1 offers a compelling, albeit still exploratory, link between mechanistic insight and clinical application. As a potential biomarker, Cav-1 may complement existing diagnostic tools by reflecting aspects of endothelial dysfunction, neuroinflammation, and BBB integrity that are not fully captured by current modalities such as MRI or cerebrospinal fluid analysis. Its context-dependent expression also suggests utility in disease monitoring, where longitudinal changes could provide insight into disease activity, progression, or therapeutic response—particularly considering the marked heterogeneity of MS. However, the absence of standardized assays and variability across studies currently limit its clinical applicability.

Therapeutically, Cav-1 represents a promising but complex target. Its central position at the intersection of immune signaling, vascular regulation, oxidative stress, and autophagy raise the possibility of multi-dimensional intervention strategies. Evidence from related disease models suggests that modulating Cav-1 could yield beneficial effects, such as enhancing BBB stability or reducing inflammatory signaling. Yet, its cell-specific and environment-dependent functions pose a significant challenge, as non-selective modulation may produce unintended or even opposing outcomes. This necessitates the development of precision-based approaches, including cell-targeted delivery systems and stage-specific interventions.

Overall, Cav-1 holds significant promise as a multifunctional biomarker and therapeutic node, but its clinical utility in MS remains to be fully established. Future studies integrating molecular, clinical, and longitudinal data will be essential to clarify its role and determine whether it can be effectively leveraged for diagnosis, disease monitoring, and targeted therapy in MS. Moreover, potential manipulation of the *CAV1* gene as a therapeutic intervention may also be of future consideration.

## Figures and Tables

**Table 1:** Summary of Cav-1 Functions and Associated Pathways in MS.

Pathway	Role of Cav-1	Key Mechanisms/Genes	Net Effect
Inflammation	Modulates immune signaling	NF-κB, cytokines (TNF-α, IL-6)	Pro- or anti-inflammatory
BBB Integrity	Regulates endothelial permeability	Tight junction proteins (occludin, claudins)	Protective or disruptive
Oxidative Stress	Influences ROS generation and signaling	eNOS, NADPH oxidase	Protective or damaging
Autophagy	Regulates autophagic flux and cell survival	LC3, Beclin-1	Adaptive or dysfunctional
Signal Transduction	Scaffold for multiple signaling pathways	MAPK, PI3K/Akt	Context-dependent modulation

This table summarizes the pleiotropic roles of Cav-1 across key biological pathways implicated in MS pathophysiology.

## Data Availability

No new data were created or analyzed in this study. Data sharing is not applicable to this article.
